# SNPMap—An integrated visual SNP interpretation tool

**DOI:** 10.3389/fgene.2022.985500

**Published:** 2022-08-19

**Authors:** Miaosen Liu, Jian Yang, Huilong Duan, Lan Yu, Dingwen Wu, Haomin Li

**Affiliations:** ^1^ School of Medicine, Zhejiang University, Hangzhou, China; ^2^ The College of Biomedical Engineering and Instrument Science, Zhejiang University, Hangzhou, China; ^3^ The Children’s Hospital, Zhejiang University School of Medicine, National Clinical Research Center, Hangzhou, China

**Keywords:** single-nucleotide polymorphism, precision medicine, visualization, variant interpretation, web application

## Abstract

New technologies, such as next-generation sequencing, have advanced the ability to diagnose diseases and improve prognosis but require the identification of thousands of variants in each report based on several databases scattered across places. Curating an integrated interpretation database is time-consuming, costly, and needs regular update. On the other hand, the automatic curation of knowledge sources always results in overloaded information. In this study, an automated pipeline was proposed to create an integrated visual single-nucleotide polymorphism (SNP) interpretation tool called SNPMap. SNPMap pipelines periodically obtained SNP-related information from LitVar, PubTator, and GWAS Catalog API tools and presented it to the user after extraction, integration, and visualization. Keywords and their semantic relations to each SNP are rendered into two graphs, with their significance represented by the size/width of circles/lines. Moreover, the most related SNPs for each keyword that appeared in SNPMap were calculated and sorted. SNPMap retains the advantage of an automatic process while assisting users in accessing more lucid and detailed information through visualization and integration with other materials.

## Introduction

Precision medicine is a novel medical approach that customizes healthcare delivery by performing diagnostic tests, especially genetic sequencing, and analyzing results to select compatible therapies and treatment plans, rather than performing a general treatment solution on a large number of patients with varying conditions (Collins and Varmus, 2015; Friedman et al., 2015; Carrasco-Ramiro et al., 2017). In recent years, genetic testing has become more prevalent and advanced in the clinical setting due to the rapid development of precision medicine. Progress in high-throughput sequencing technologies, particularly next-generation sequencing (NGS), has dramatically improved their applicability across different fields, including hereditary cancer, pediatrics, and cardiovascular, aiming to diagnose diseases, predict drug reactions, and select treatment options (Friedman et al., 2015; Stavropoulos et al., 2016; Nakagawa and Fujita, 2018; Zhang et al., 2020).

On the other hand, the application of whole-exome sequencing (WES) and whole-genome sequencing (WGS) implies that each sequencing could see 200,000–400,000 WES variants or 3,900,000 WGS variants recognized on each subject (Yang et al., 2013; Lionel et al., 2018), and hundreds of clinical variants with potential clinical significance remain even after multistage filtering (Zhang et al., 2020). Even after interpreting the variants, ambiguities and inaccuracies can still occur in the interpretation notes (McCarthy et al., 2014; Wenger et al., 2017). While recognizing a large number of variants is a critical milestone in sequencing technology efforts to accurately interpret have become a considerable obstacle to high-quality clinical genetic reporting (Zhang et al., 2020), restricting the development of precision medicine (Good et al., 2014).

Researchers have worked hard over the last few years to develop accurate, rapid, and cost-effective technologies or protocols for variant analysis and interpretation, yielding several distinct approaches. The American College of Medical Genetics and Genomics (ACMG) and the Association for Molecular Pathology (AMP) have already published recommendations for standards and guidelines in sequence variant interpretation, with detailed rules on evidence, direction, and strength classification (Richards et al., 2015). For example, to achieve the classification of pathogenic/benign variants, four weight levels (“supporting”, “moderate”, “strong”, and “very strong”) have been created for pathogenic criterion, while two weight levels (“supporting”, “strong”) have been created for benign criterion. Creating manually curated sequence variants interpretation databases is a popular approach. ClinVar (Landrum et al., 2018), dbSNP (Sherry et al., 2001; Kitts et al., 2014), and SNPedia (Cariaso and Lennon, 2011) are the databases with expert-curated content or community-maintained knowledge and variant interpretations (Allot et al., 2018). However, manually curated interpretations necessitate an expert review of each of these variations individually, which is a time-consuming, costly, and arduous task. As a result, manually curated interpretations often have a limited scale and cannot keep up with increasing domain knowledge. Furthermore, despite the guidelines developed for variant interpretation, there are still degrees of subjectivity and uncertainty that can lead to inconsistent classification across different laboratories (Balmaña et al., 2016; Harrison et al., 2017; Kim et al., 2019).

Automatically curated databases and tools are developed to compensate for these shortcomings. LitVar (Allot et al., 2018) is a powerful semantic search engine for variant information that addresses issues faced by manually curated tools. It collects biomedical literature related to a variant using PubMed and PubTator tools while also utilizing advanced text mining techniques to compute and extract entities such as diseases and chemicals that are linked to the variant. It has the advantage of being automatic, broad, and up to date. However, the information provided by LitVar on each variant/SNP is a traditional literature list that requires the user to read and understand large sections of the paragraph.

Among these approaches, integrated visualization of information in an automatically updated and curated database is considered a missing piece of the whole process. Furthermore, additional valuable data could be further generated from integrated curated data. Therefore, SNPMap is developed to facilitate better understanding and comprehension of single-nucleotide polymorphisms (SNPs), the most common type of genetic variations.

## Materials and methods

**FIGURE 1:**
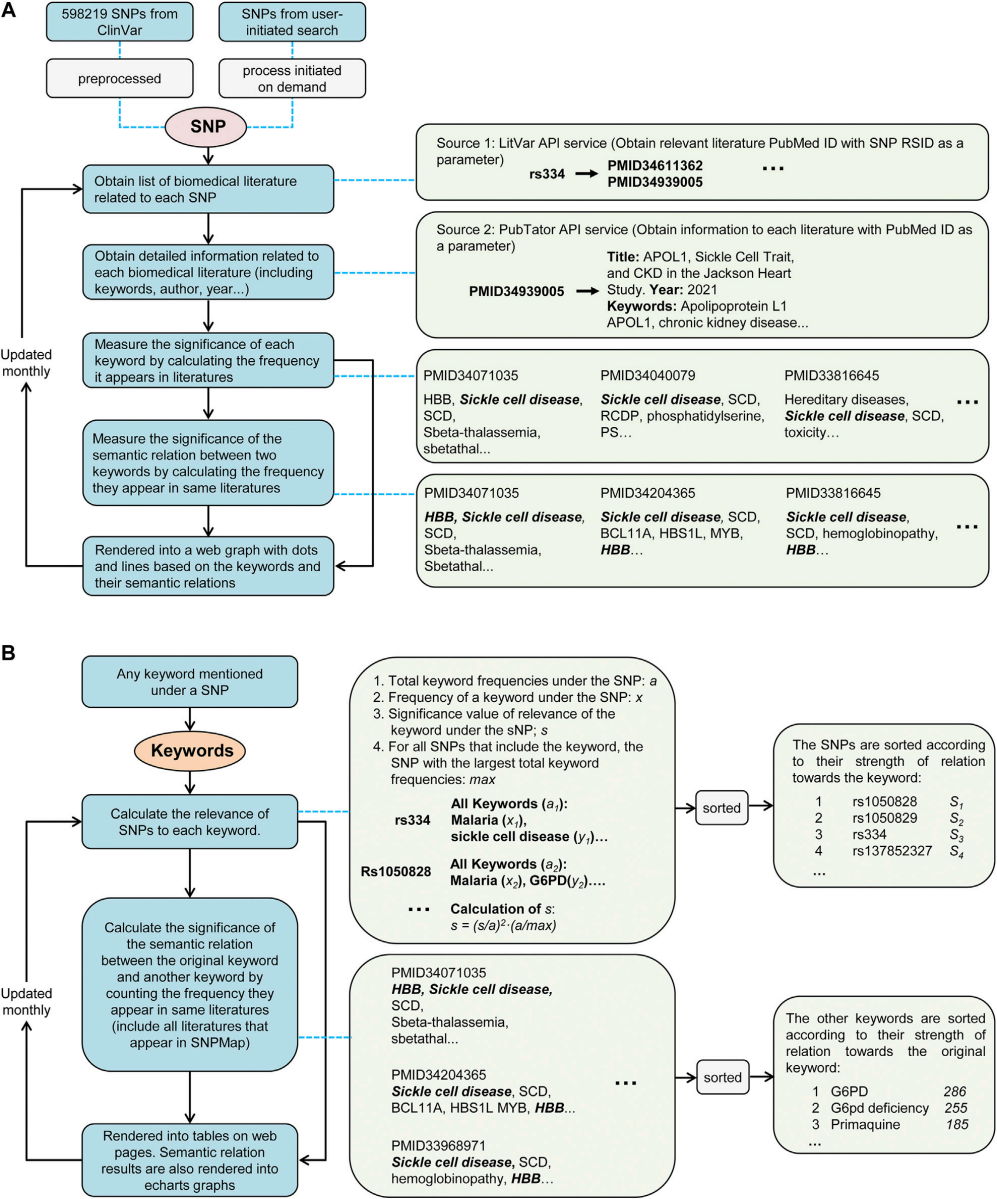
The data processing workflow of SNPMap. **(A)** The process of obtaining keyword information and rendering graph from SNPs. **(B)** The process of calculating SNP or external keyword connections of keywords mentioned in SNPMap.

The primary reference SNPs were obtained from the ClinVar using application programming interfaces (API). These SNPs were selected for their possible relevance to large amounts of biomedical literature, ensuring the ability to build a preprocessed database with sufficient information. When a user initiates a search with SNP that is not pre-selected in SNPMap, an online workflow (Figure 1) will query relevant information to build and add new knowledge content to SNPMap in real-time. This workflow obtains a list of relevant literature for a specific SNP through a LitVar API query. Utilizing the PubMed identifiers (PMIDs) of these literature, literature-related details, such as abstracts, keywords, etc., are subsequently obtained by accessing PubTator’s online service. These SNP-related keywords are divided into three categories (data, genes, and chemicals) based on PubTator data. Before further using these keywords, the form of these keywords was standardized using a python module called LemmInflect (Available from: https://lemminflect.readthedocs.io).

The significance of the association between a keyword and SNP depends on how often the keyword appears in all relevant literature, and this significance was assessed by the frequency of the two keywords appearing together in the same document. In the visualization of an SNP, keywords were used to generate network nodes, where the node size reflects the significance of the association between keywords and SNPs, and the thickness of the connecting line between nodes reflects the significance of the association between the two keywords. Since a keyword can be associated with multiple SNPs in different works of literature, through the quantitative assessment of the significance of keyword and SNP associations, we also obtained the ranking information of the SNPs and other keywords corresponding to a keyword, which provides a basis for retrieving SNPs and other keywords by keyword. The database of SNPMap is designed to update monthly. Node.js was used to develop the website, and the visualization was based on Apache ECharts (https://echarts.apache.org/). Other calculations and analysis modules used are implemented via Python.

## Results

### The visualization of single-nucleotide polymorphism-associated keywords

**FIGURE 2:**
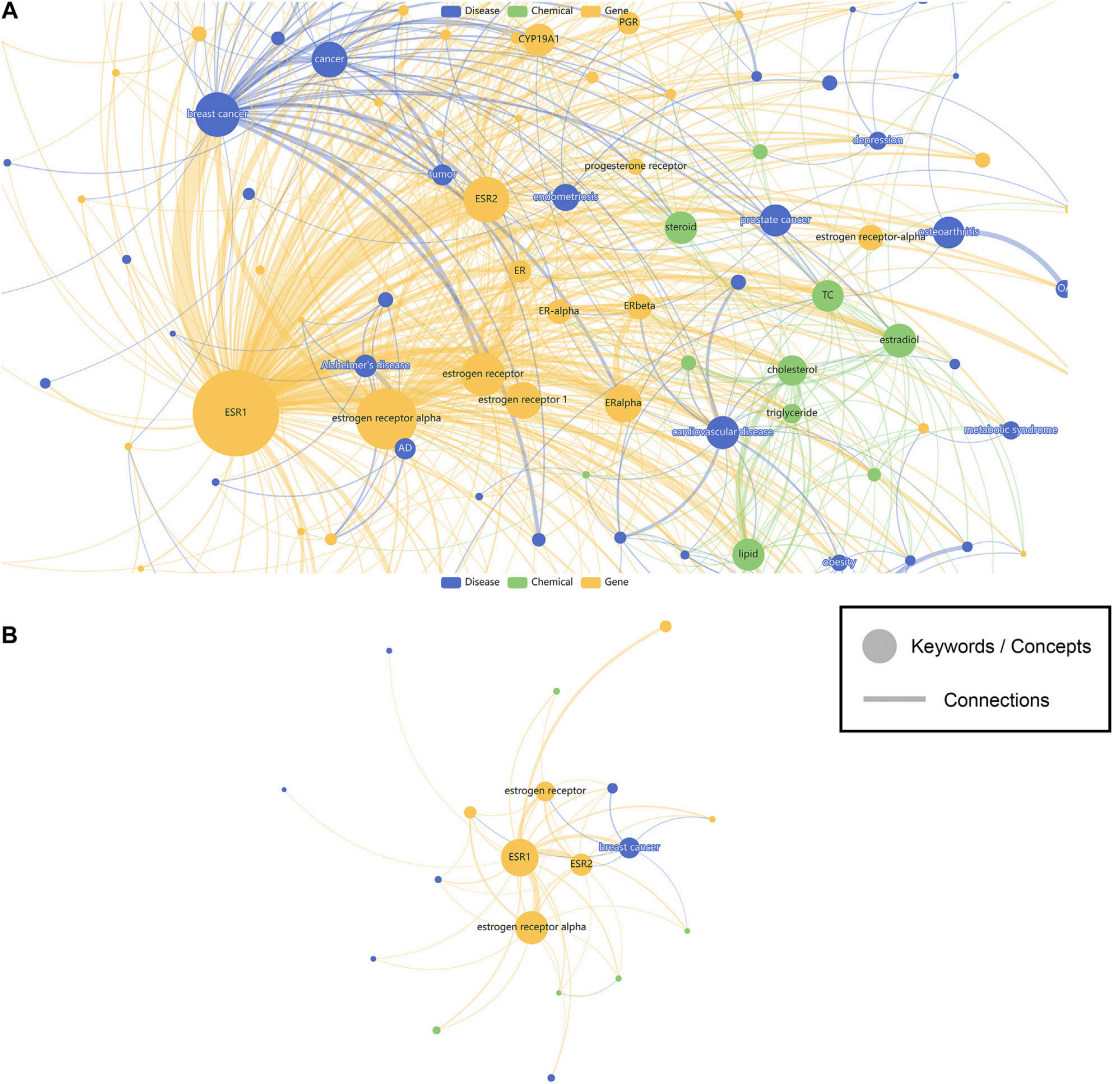
The visualization of SNP associated keywords. **(A)** Show all keywords. **(B)** Show less keywords. Nodes represent keywords while edges represent connections. Sizes of the nodes represent the significance of keywords, while width of the edges represent the strength of connections. Colors of the nodes are assigned based on the different categories (genes, diseases, chemicals) of the keywords.

In this study, 598219 SNPs recorded with ClinVar information have their related biomedical literature accessed through LitVar and PubTator. A total of 789115 keywords were identified, and their most relevant SNPs in biomedical literature were counted and sorted. A website called SNPMap was published online (http://snp.nbscn.org) for users to explore these millions of associations. First, users can query SNP-related data and visualize all the association information by entering the dbSNP Reference SNP identifier (RSID or RefSNP ID) or the HGVS notation of the SNP in the search field. Visualization of an SNP (rs2234693) is taken as an example (Figure 2). The node’s color reflects the keyword type, the node’s size represents the significance of the association with that SNP, and the thickness of the line linking the nodes represents the association of the two keywords. Since the research degree of different SNPs varies greatly, some SNPs with thousands of research reports can form a very complex visualization network (Figure 2A). For this reason, SNPMap provides a function to adjust the filtering keywords dynamically, and users can adjust the complexity of the visualization network as needed (Figure 2B). Another visualization layout called Circular Map could show the keywords in a more organized way. In both layouts, keywords and semantic relations could be highlighted by hovering the mouse cursor on them. Double-clicking on a keyword will display a new page showing only the information associated with that keyword in that SNP.

### Relevant literature, keywords, genome-wide association studies (GWAS) studies, and distribution in different populations are further presented on the SNPMap

In addition to visualizing the network, relevant literature, keywords, GWAS, and distribution in different populations are further presented on the SNPMap through tables. SNPMap also allows the use of concept keywords to retrieve information and sort the list of realistically associated SNPs in addition to displaying keyword-associated concepts.

**FIGURE 3:**
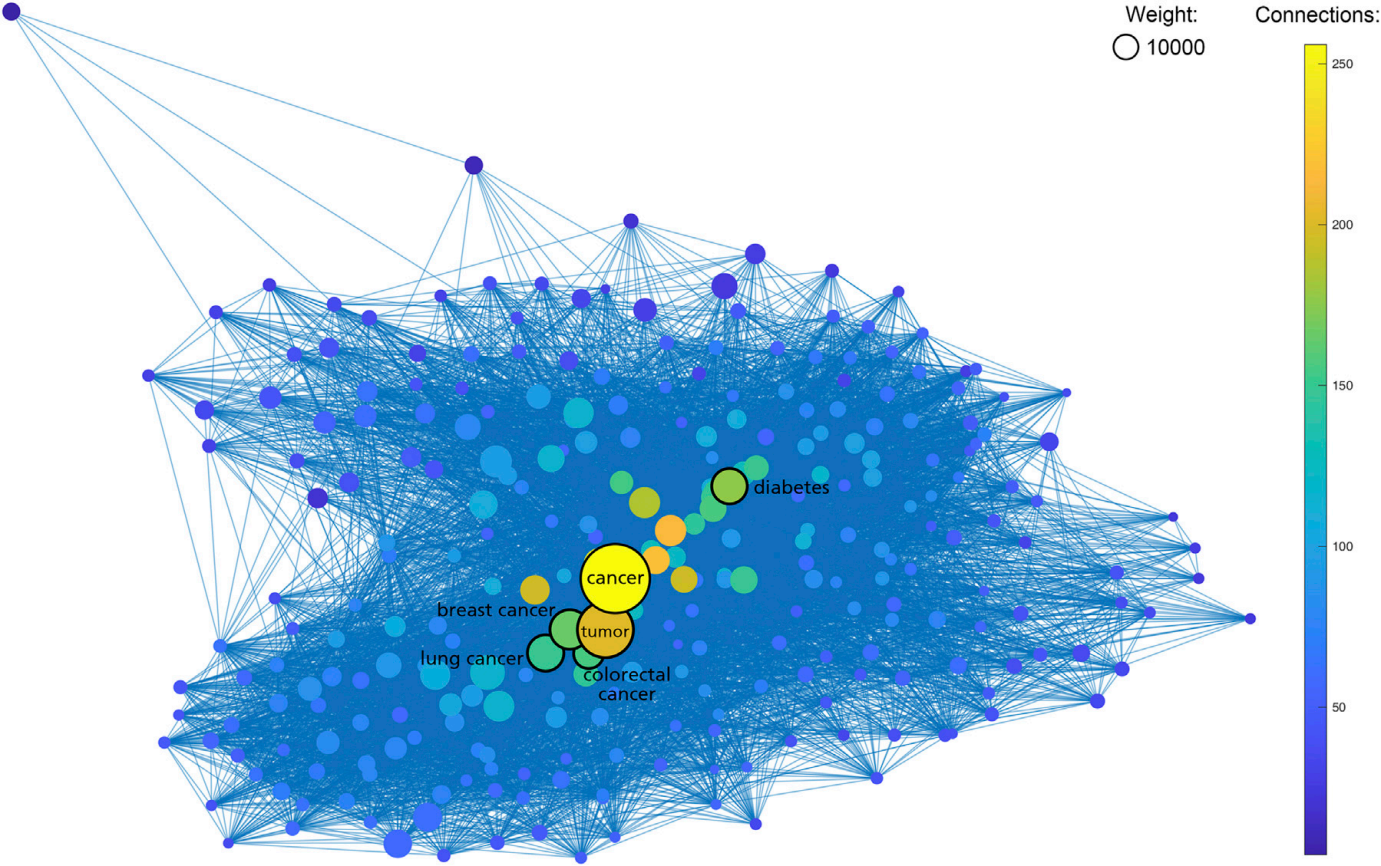
Graph containing 654 SNPMap keywords (nodes) and their internal connections (edges). Keywords with less ten total counts or 50 connections are excluded. Sizes of nodes represent the total counts of keywords. Colors of nodes represent the numbers of connections to keywords.

As shown in Table 1, the most frequent keyword in SNPMap is “cancer”, with 48412 SNPs having this word as one of their keywords. Many of the other top keywords are also cancer-related, e.g., “breast cancer”, “tumor”, “BRCA1”, “BRCA2”, “colorectal cancer”, “TP53”, and “EGFR”. Other top keywords include “diabetes”, “Alzheimer’s disease”, “toxicity”, “Parkinson’s disease” etc. When rendering the more prominent SNPMap keywords and connections among them in a graph, the similar keywords, especially ones related to cancer, are shown as more significant (Figure 3). This is partly a reflection of what is heated in biomedical research.

**TABLE 1:** Most frequent keywords in SNPMap.

Keyword	Frequency
Cancer	48412
Tumor	35927
Breast cancer	20082
BRCA1	12951
BRCA2	11636
Diabetes	11312
TP53	10779
Colorectal cancer	9984
Lipid	9962
Alzheimer’s disease	9851
EGFR	8442
AD	8008
Toxicity	7852
Parkinson’s disease	7733
Inflammation	7302
BRAF	7178
KRAS	7172
Hypertension	6968
Lung cancer	6879
Cholesterol	6672

### Efficacy comparison between SNPMap and ClinVar

**FIGURE 4:**
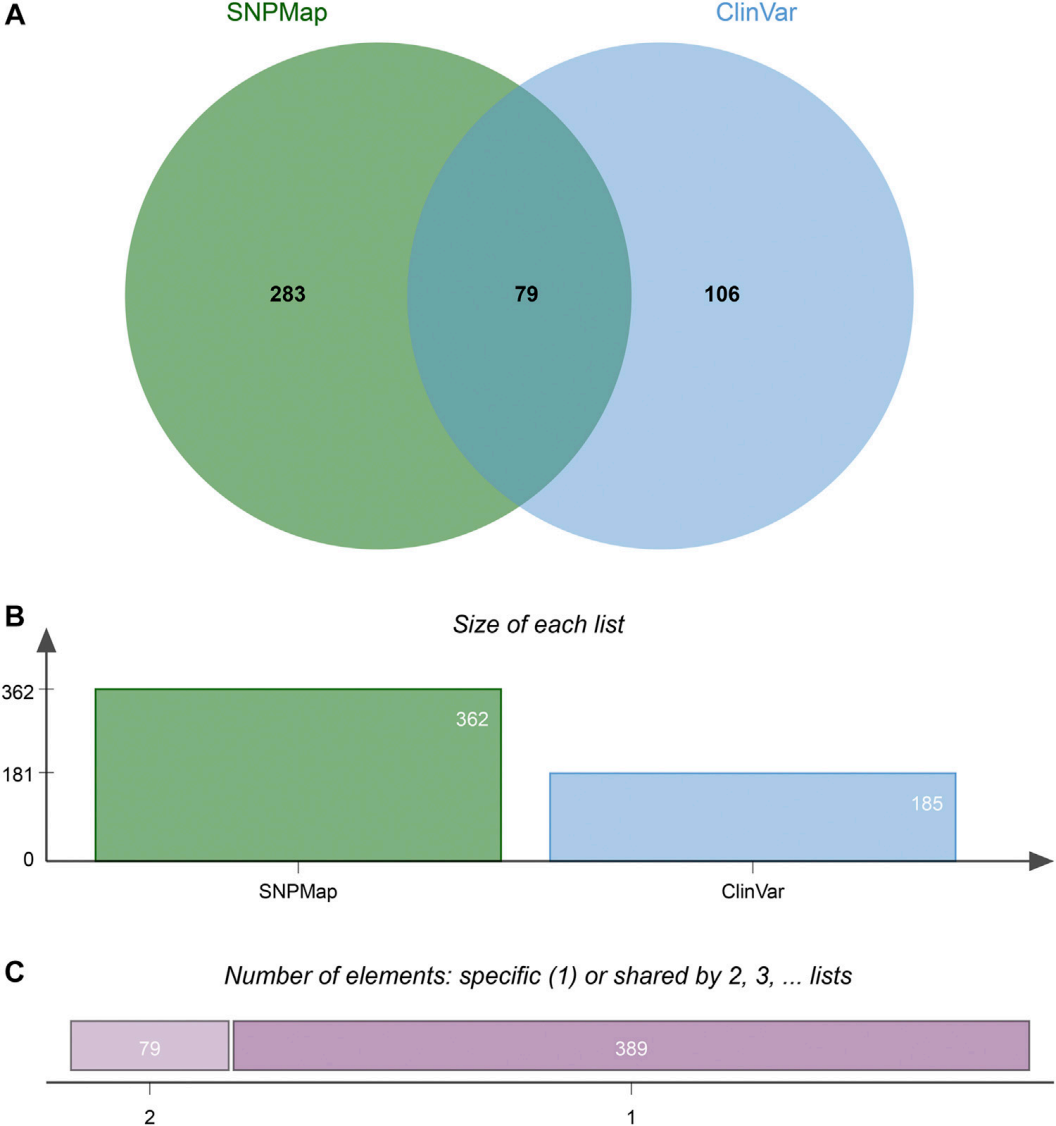
The difference of keywords between SNPMap and ClinVar of 100 random selected SNPs. **(A)** Numbers of keywords mentioned only in SNPMap, only in ClinVar, or in both. **(B)** Numbers of keywords mentioned in SNPMap, ClinVar. **(C)** Numbers of keywords mentioned in only one, or both platforms. The Venn diagram is generated with jvenn (Bardou et al., 2014).

A total of 100 SNPs were randomly selected to compare the efficacy of SNPMap and ClinVar, and the differences between SNPMap and ClinVar were compared in terms of keywords, as shown in the Venn diagram (Figure 4). A few selected comparisons are listed in Table 2. Since LitVar information only contains diseases, only results related to diseases are selected from SNPMap for comparison. It could be observed that under many circumstances, SNPMap has more coverage of the concepts related to each SNP. Among all the concepts mentioned under the 100 SNPs by the platforms, 283 concepts are mentioned in SNPMap, 106 concepts are mentioned in LitVar, and 79 concepts are mentioned in both SNPMap and LitVar (Figure 4).

**TABLE 2:** Some selected SNPs and comparisons of their concepts under SNPMap and ClinVar.

	SNPMap (diseases)	ClinVar
rs146632606	*Gitelman syndrome*, Monogenic urinary stone disease, hyperoxaluria, atherosclerosis, hypertension, hypotension, hypocalciuria, secondary hyperaldosteronism	*Gitelman syndrome*
rs7482144	*Breast/ovarian cancer*, cutaneous melanoma, melanoma	*Breast/ovarian cancer*, hereditary cancer-predisposing syndrome
rs80358086	*Breast cancer, cancer*	*Breast cancer, cancer*, hereditary cancer-predisposing syndrome, Hereditary breast and ovarian cancer syndrome, ovarian cancer
rs137853334	*Diabetes mellitus*, hepatocellular carcinoma, Congenital hyperinsulinism, hyperinsulinaemic hypoglycaemia, hyperglycemia, hypoglycaemia	*Diabetes mellitus*
rs199498900	*Walker-warburg syndrome, limb girdle muscular dystrophy, congenital muscular dystrophy*, brain anomaly, ocular abnomality	*Walker-warburg syndrome, limb girdle muscular dystrophy, congenital muscular dystrophy*
rs111656822	*Epilepsy*, carnitine deficiency, idiopathic generalized epilepsy	*Epilepsy*, Epilepsy with grand mal seizures on awakening, Leukoencephalopathy with ataxia

### Efficacy comparison between SNPMap and LitVar

**FIGURE 5:**
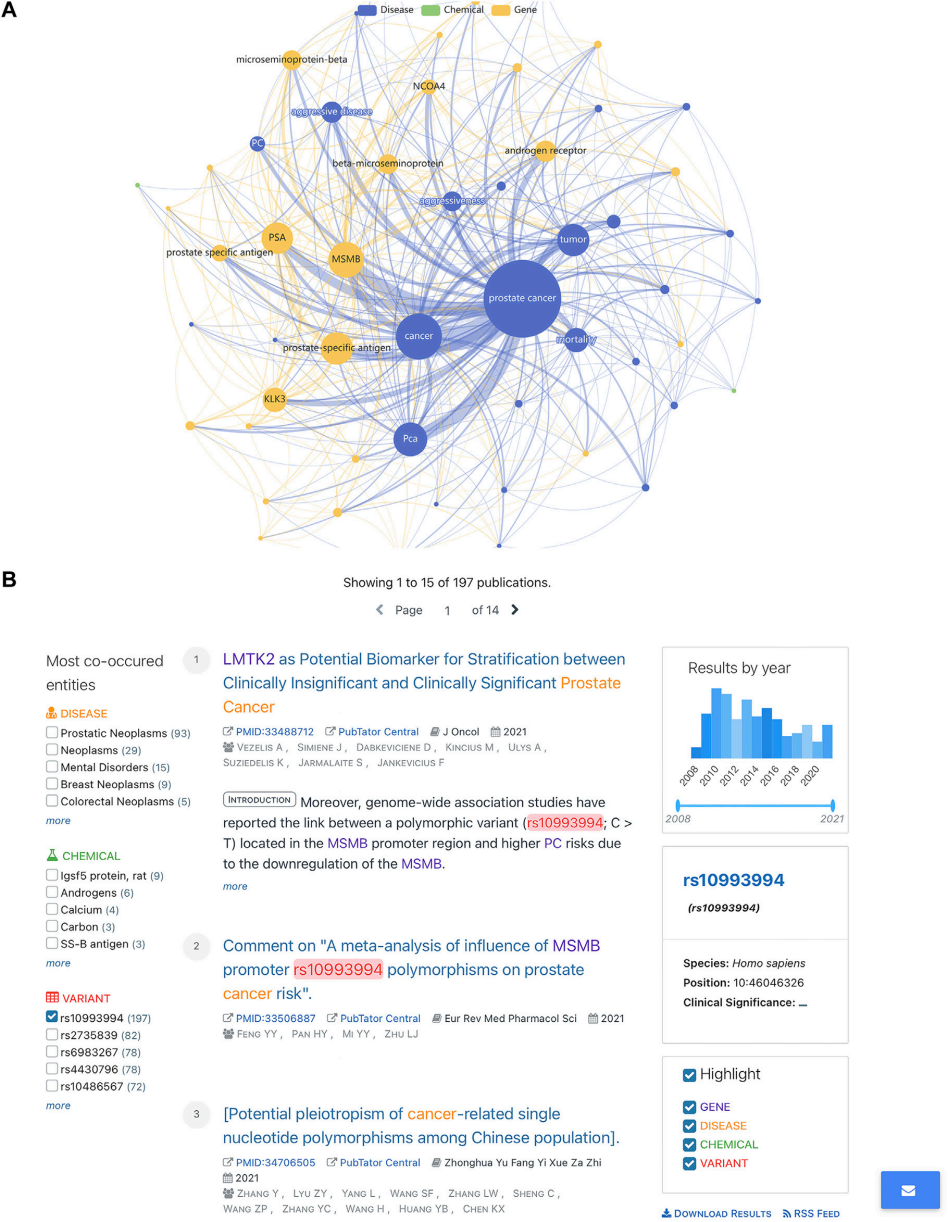
Comparing SNPMap with LitVar. **(A)** rs10993994 in SNPMap. **(B)** rs10993994 in LitVar.

When the results of SNPMap were compared with another automatic-curated LitVar, the advantage of SNPMap’s intuitive visualization could be established. When interpreting the possible implications of SNPs on LitVar, the main user interface that the user encounters are biomedical literature pages (Figure 5); a few selected comparisons are listed in Table 3. Because both LitVar and SNPMap’s data are mainly obtained from PubTator, there are similarities between LitVar’s and SNPMap’s main keywords. Furthermore, it could be concluded that LitVar’s keywords are more concise and precise, while SNPMap’s keywords are often expanded and supplemented based on the most important keywords (Table 3).

**TABLE 3:** Some selected SNPs and comparisons of their concepts under LitVar and SNPMap.

	SNPMap (Top keywords)	LitVar (Top keywords)
rs10993994	Diseases: Prostatic Neoplasms (93), Neoplasms (29), Mental Disorders (15), Breast Neoplasms (9), Colorectal Neoplasms (5)	Prostate cancer (228), Cancer (89), MSMB (43), PCA (35), Prostate-specific antigen (29), Tumor (28), PSA (25), KLK3 (16), Mortality (16), Androgen receptor, (15) etc
	Chemicals: Igsf5 protein, rat (9), Androgens (6), Calcium (4), SS-B antigen (3), Carbon (3)	
rs334	Diseases: Sickle Cell Anemia (81), Systemic carnitine deficiency (48), Malaria (43), Anemia (40), Thalassemia (16), Genetic Diseases, Inborn (13)	Malaria (93), Sickle cell disease (91), HBB (53), Anemia (45), SCD (44), Thalassemia (36), Stroke (27), Mortality (26), Hydroxyurea (21), Infection, (21) etc
	Chemicals: Glutamic Acid (13), Valine (10), Valine-Valine-Saquinavir (9), Oxytocin, Glu (4)- (8), Adenine (5)	
rs7903146	Diseases: Diabetes Mellitus (507), Type 2 Diabetes Mellitus (237), Obesity (130), Glucose Intolerance (40), Stroke (35)	Diabetes (692), TCF7L2 (556), Type 2 Diabetes (382), Glucose (303), Insulin (251), Transcription factor 7-like 2 (206), Obesity (154), Diabetic (134), Diabetes mellitus (127), Type 2 Diabetes Mellitus, (92) etc
	Chemicals: Glucose (195), Cholesterol (41), Triglycerides (40), Metformin (27), Carbohydrates (22)	
rs112445441	Diseases: Colorectal Neoplasms (510), Neoplasms (451), Adenomatous Polyposis (59), Carcinoma, Non-Small-Cell (47), Melanoma (44)	KRAS (1198), Cancer (1084), Tumor (1030), Colorectal cancer (1018), EGFR (490), BRAF (466), CRC (386), NRAS (266), PIK3CA (240), Epidermal Growth Factor Receptor, (237) etc
	Chemicals: AT 61 (53), Cetuximab (52), Guanosine Triphosphate (41), Glycine (32), irinotecan (25)	
rs121913500	Diseases: Glioma (774), Neoplasms (678), Glioblastoma (340), Astrocytoma (291), Oligodendroglioma (228)	Glioma (1328), Tumor (1307), Glioblastoma (890), Cancer (842), IDH1 (578), IDH (504), GBM (290), Brain tumor (246), Astrocytoma (242), IDH1/2, (184) etc
	Chemicals: Alpha-hydroxyglutarate (144), Isocitrates (144), Arginine Vasopressin (72), Activated-Leukocyte Cell Adhesion Molecule (67), Histidine-pyridine-histidine-3 (55)	

## Discussion

In this study, we developed an integrated visual SNP interpretation tool—SNPMap. SNPMap periodically obtains and updates SNP-related information from LitVar, PubTator, and GWAS Catalog API tools, and data is processed for extraction, integration, and visualization before users’ access. For each SNP, two graphs are generated to describe keywords and their semantic relations. We have also calculated and sorted the most related SNPs for keywords on SNPMap. Finally, SNPMap can combine the advantages of an automatic process with the benefits of visualization. When compared to manually curated SNP interpretation knowledge bases such as ClinVar, SNPMap lacks some expert-annotated information (e.g., pathogenic, benign, etc.) but can provide more association information, which comprises some associations with insufficient evidence or inconsistent prevailing results, which has more implications for clinical discovery and identification of novel variant-disease associations.

As shown in Table 1, seven of the top 10 entities listed on SNPMap are closely related to cancer topics, with neurodegenerative diseases, cardiovascular diseases, and metabolic diseases accounting for a significant chunk of those top entities. It is possible that SNPMap tools have adequate usability under disease-variant research environments, predominantly cancer research, and would be a valuable tool in acquiring information on past variant studies and exploring potential variants for further research. It demonstrates SNPMap’s ability to facilitate research and knowledge into various diseases.

In many cases, SNPMap outperforms ClinVar regarding coverage of the concepts associated with each SNP. As shown in Figure 4, among all the concepts mentioned under the 100 SNPs by the platforms, some are only mentioned in SNPMap or ClinVar, while a significant number are mentioned in both SNPMap and ClinVar. Among the 100 SNPs, over half of them contain concepts that are mentioned by both platforms. Many of the 283 concepts mentioned exclusively by SNPMap are distinctive concepts not mentioned by ClinVar under the same SNP, with some of the concept connections confirmed by the recent biomedical literature, while some others are extended from a concept mentioned by both platforms (e.g., symptoms of a medical syndrome). Although ClinVar has fewer exclusive concepts covered than SNPMap, it provides information about whether an SNP variant is linked to a disease and contains clinical significance information that is not included in SNPMap (pathogenic, benign, etc.). The reasons for less coverage may be attributed to the following factors. Firstly, ClinVar information is maintained by human examiners, which limits the scope and timeliness of that information and necessitates time-consuming efforts to verify each connection, while SNPMap’s automation process allows for a more comprehensive collection of concepts from the latest literatures without human factors. Secondly, SNPMap contains and calculates keywords from biomedical literature related to each SNP, which may include keywords that are trivially mentioned in the literature, resulting in the inflated numbers of keyword mentions in some cases. In conclusion, the comparison of two services has confirmed the ability of SNPMap to cover significant concepts mentioned by the human-managed peer services while extending into more concepts that include the latest developments and related symptoms.

Because both LitVar and SNPMap’s data get their data from PubTator, there are some similarities between LitVar and SNPMap’s main keywords. When comparing SNPMap results to LitVar, the advantage of SNPMap’s intuitive visualization could be established. As shown in Table 3, LitVar keywords are more concise and precise, while SNPMap’s keywords are often expanded and supplemented based on the most important keywords. While some keywords related to disease, chemicals, and variants are displayed on the sidebar of the web page, the location made the keywords less intuitive and precise, while missing out on some important concepts (e.g., prostate-specific antigen (PSA) for rs10993994, since it is previously reported association of the SNP (Wiklund et al., 2009; Wang et al., 2021)). The graph on SNPMap provides additional information that is not represented on LitVar that includes keyword connections that have strong connections among concepts of “prostate cancer”, and “MSMB” (the gene where the SNP is located), “prostate-related antigen” (closely related to prostate cancer).

In addition, SNPMap offers a reverse search—using concepts as keywords to find SNPs and other concepts that are prevalent in related biomedical literature, a feature that is not available in dbSNP, LitVar, or any other tools. Using the concept “breast cancer” as an example (http://snp.nbscn.org/word/breast%20cancer), related SNPs are highlighted by SNPMap with many of them having high beta and *p* values in previous GWAS studies related to breast cancer. It allows users to quickly navigate to the page that corresponds to the corresponding keyword.

While SNPMap has been a comprehensive tool for providing variants and concepts with background information, the information it delivers should be considered under the condition that maximum information sources come from biomedical literature. Thus, the results should be interpreted as a general representation of the variant or concept in biomedical literature. The standard of different biomedical literature varies, and the quality and quantity of related biomedical literature will impact the contents of the results. While relations mentioned in more literature are more likely to be valid, no relation could be considered 100% certain. Besides, any possible relations that are not mentioned in previous biomedical literature will not be displayed, so the results displayed should be viewed as retrospective, and even though novel connection discoveries could be promptly added to the SNPMap database, the tool is questionable to be used for finding brand new relations.

Another drawback of SNPMap is its limited scale. The study only calculated 598,219 SNPs included in ClinVar, against more than a billion SNPs on dbSNP. The limited scale of SNPs that include enough biomedical literature information to render a keyword graph further cut the size to 46,747 SNPs that could reach the threshold we installed to generate graphs, although less biomedical literature reflects that less research has been performed on the SNPs, implying that researchers are less likely to have the interest to request information into those SNPs. To compensate for the disadvantages brought by the limited scale of the database, we calculate immediate SNP data demands made by our users, which composed of are SNPs that are not yet stored inside our database, in real-time. Users will be notified that the calculation will be completed from a few seconds to a few minutes and will be able to read the data instantly after completion of the calculation.

As SNPMap currently stands, the web application has become a useful tool with vast potentials for researchers and clinical practitioners alike. For researchers, SNPMap could be useful in obtaining a thorough picture about how a variant was researched in the past, leading the directions of future research topics on the variant. With the additional resources of concept connections, researchers would also be able to identify significant variants related to diseases, organs, cells etc., thus finding variants for their own research projects. SNPMap could even potentially help researchers dig out new information between SNPs and concepts that were buried in large amounts of biomedical literatures. For practitioners, the tool could be helpful in facilitating quick interpretation and filtration of huge variant datasets, helping practitioners to distinguish variants with significance efficiently, and saving precious time in preparing clinical genetic reports.

## Conclusion

A user-friendly, visualized and automatically curated SNP interpretation tool called SNPMap was proposed and developed in this study which has applications in several scenarios, including interpretation of clinical testing results and scientific research outcomes, especially under disease-variant research environments. For researchers, it would be a valuable tool in acquiring information on past variant studies, exploring potential variants for further research, and for clinical practitioners, it could be extremely useful in interpreting and reporting genetic testing results with large amounts of variant information. The database will be regularly updated with new SNP/variant information since new biomedical literature works are being published incessantly.

## Data Availability

The original contributions presented in the study are included in the article/supplementary material, further inquiries can be directed to the corresponding author.

## References

[B1] Allot A. Peng Y. Wei C. H. Lee K. Phan L. Lu Z. (2018). LitVar: a semantic search engine for linking genomic variant data in PubMed and PMC. Nucleic Acids Res. 46 (W1), W530–W536. 10.1093/nar/gky355 29762787 PMC6030971

[B2] Balmaña J. Digiovanni L. Gaddam P. Walsh M. F. Joseph V. Stadler Z. K. (2016). Conflicting interpretation of genetic variants and cancer risk by commercial laboratories as assessed by the prospective registry of multiplex testing. J. Clin. Oncol. 34 (34), 4071–4078. 10.1200/JCO.2016.68.4316 27621404 PMC5562435

[B3] Bardou P. Mariette J. Escudié F. Djemiel C. Klopp C. (2014). jvenn: an interactive Venn diagram viewer. BMC Bioinforma. 15 (1), 293. 10.1186/1471-2105-15-293 PMC4261873 25176396

[B4] Cariaso M. Lennon G. (2011). SNPedia: a wiki supporting personal genome annotation, interpretation and analysis. Nucleic Acids Res. 40 (D1), D1308–D1312. 10.1093/nar/gkr798 22140107 PMC3245045

[B5] Carrasco-Ramiro F. Peiró-Pastor R. Aguado B. (2017). Human genomics projects and precision medicine. Gene Ther. 24 (9), 551–561. 10.1038/gt.2017.77 28805797

[B6] Collins F. S. Varmus H. (2015). A new initiative on precision medicine. N. Engl. J. Med. 372 (9), 793–795. 10.1056/NEJMp1500523 25635347 PMC5101938

[B7] Friedman A. A. Letai A. Fisher D. E. Flaherty K. T. (2015). Precision medicine for cancer with next-generation functional diagnostics. Nat. Rev. Cancer 15 (12), 747–756. 10.1038/nrc4015 26536825 PMC4970460

[B8] Good B. M. Ainscough B. J. McMichael J. F. Su A. I. Griffith O. L. (2014). Organizing knowledge to enable personalization of medicine in cancer. Genome Biol. 15 (8), 438. 10.1186/s13059-014-0438-7 25222080 PMC4281950

[B9] Harrison S. M. Dolinsky J. S. Knight Johnson A. E. Pesaran T. Azzariti D. R. Bale S. (2017). Clinical laboratories collaborate to resolve differences in variant interpretations submitted to ClinVar. Genet. Med. 19 (10), 1096–1104. 10.1038/gim.2017.14 28301460 PMC5600649

[B10] Kim Y. E. Ki C. S. Jang M. A. (2019). Challenges and considerations in sequence variant interpretation for mendelian disorders. Ann. Lab. Med. 39 (5), 421–429. 10.3343/alm.2019.39.5.421 31037860 PMC6502951

[B11] Kitts A. Phan L. Ward M. Holmes J. B. (2014). “The database of short genetic variation (dbSNP),” in The NCBI handbook [internet]. 2nd edition (Bethesda, MD: National Center for Biotechnology Information-US).

[B12] Landrum M. J. Lee J. M. Benson M. Brown G. R. Chao C. Chitipiralla S. (2018). ClinVar: improving access to variant interpretations and supporting evidence. Nucleic Acids Res. 46 (D1), D1062–D1067. 10.1093/nar/gkx1153 29165669 PMC5753237

[B13] LemmInflect (2021). A python module for English word lemmatization and inflection. [Online]. Available at: https://lemminflect.readthedocs.io/en/latest/ (Accessed 16 10, 2021).

[B14] Lionel A. C. Costain G. Monfared N. Walker S. Reuter M. S. Hosseini S. M. (2018). Improved diagnostic yield compared with targeted gene sequencing panels suggests a role for whole-genome sequencing as a first-tier genetic test. Genet. Med. 20 (4), 435–443. 10.1038/gim.2017.119 28771251 PMC5895460

[B15] McCarthy D. J. Humburg P. Kanapin A. Rivas M. A. Gaulton K. Cazier J.-B. (2014). Choice of transcripts and software has a large effect on variant annotation. Genome Med. 6 (3), 26. 10.1186/gm543 24944579 PMC4062061

[B16] Nakagawa H. Fujita M. (2018). Whole genome sequencing analysis for cancer genomics and precision medicine. Cancer Sci. 109 (3), 513–522. 10.1111/cas.13505 29345757 PMC5834793

[B17] Richards S. Aziz N. Bale S. Bick D. Das S. Gastier-Foster J. (2015). Standards and guidelines for the interpretation of sequence variants: a joint consensus recommendation of the American College of medical genetics and genomics and the association for molecular Pathology. Genet. Med. 17 (5), 405–424. 10.1038/gim.2015.30 25741868 PMC4544753

[B18] Sherry S. T. Ward M.-H. Kholodov M. Baker J. Phan L. Smigielski E. M. (2001). dbSNP: the NCBI database of genetic variation. Nucleic Acids Res. 29 (1), 308–311. 10.1093/nar/29.1.308 11125122 PMC29783

[B19] Stavropoulos D. J. Merico D. Jobling R. Bowdin S. Monfared N. Thiruvahindrapuram B. (2016). Whole genome sequencing expands diagnostic utility and improves clinical management in pediatric medicine. NPJ Genom. Med. 1 (1), 15012. 10.1038/npjgenmed.2015.12 28567303 PMC5447450

[B20] Wang X. Hayes J. E. Xu X. Gao X. Mehta D. Lilja H. G. (2021). Validation of prostate cancer risk variants rs10993994 and rs7098889 by CRISPR/Cas9 mediated genome editing. Gene 768, 145265. 10.1016/j.gene.2020.145265 33122083 PMC7796996

[B21] Wenger A. M. Guturu H. Bernstein J. A. Bejerano G. (2017). Systematic reanalysis of clinical exome data yields additional diagnoses: implications for providers. Genet. Med. 19 (2), 209–214. 10.1038/gim.2016.88 27441994

[B22] Wiklund F. Zheng S. L. Sun J. Adami H.-O. Lilja H. Hsu F.-C. (2009). Association of reported prostate cancer risk alleles with PSA levels among men without a diagnosis of prostate cancer. Prostate 69 (4), 419–427. 10.1002/pros.20908 19116992 PMC3348520

[B23] Yang Y. Muzny D. M. Reid J. G. Bainbridge M. N. Willis A. Ward P. A. (2013). Clinical whole-exome sequencing for the diagnosis of mendelian disorders. N. Engl. J. Med. 369 (16), 1502–1511. 10.1056/NEJMoa1306555 24088041 PMC4211433

[B24] Zhang J. Yao Y. He H. Shen J. (2020). Clinical interpretation of sequence variants. Curr. Protoc. Hum. Genet. 106 (1), e98. 10.1002/cphg.98 32176464 PMC7431429

